# Mr. Harrup, on the Prevailing Epidemic

**Published:** 1803-06-01

**Authors:** Robert Harrup

**Affiliations:** Chobham, Surry


### *To the Editors of the Medical and Physical Journal.*

GENTLEMEN,

THE Epidemic which at present prevails in the metropolis, and in many parts of the country, made its first appearance in this place about the middle of February last, which was some time before its effects were felt in any of the adjacent parishes. The patient, at the commencement of the disorder, complained of chilliness, which was soon succeeded by a high degree of fever with violent pain of the head, generally in the forehead, sometimes in the back part, and frequently all over it, which was a never failing symptom of the disorder. The back was also considerably affected; and, to use the words of most patients, their bones ached all over. Pulse full and hard, from 85 to 100; tongue furred in the middle, with red edges; vertigo with slight delirium at times, particularly in the night; sickness which, when amounted to vomiting, brought up a bilious matter, and procured a temporary relief; costive in general, but sometimes a diarrhœa from the beginning; pains of the legs and some parts of the abdomen, most frequently of the hepatic region; in several, the whole of the abdomen was somewhat swelled and tense, with pain on the slightest pressure, especially over the pubis; urine high coloured during the continuance of fever, depositing afterwards a reddish yellow sediment; no appetite; thirst considerable; restless, inclined to doze, but disturbed by dreams. When the fever had in a great degree abated, a harrasing dry cough began, but in no instance before that time, time, and several now felt a fixed pain in some part of the chest. The young and robust were uniformly attacked suddenly; and in them the fever ran to a high degree, but they much sooner recovered health and strength than those in whom the symptoms were less severe. A few, on the first attack, discharged a frothy saliva tinged with blood, which disappeared in a short time without any particular consequences. Many, on recovering from the disorder, were affected with a dread and depression of spirits, similar to those labouring under hypochondriasis, but which soon disappeared. Several complained of vertigo while recovering, and it was observable that while this continued, their appetite did not return. Those who had been subject formerly to other diseases, seldom failed having a return, either at the commencement or departure of this epidemic. In one who had frequent threatenings of Apoplexy, it commenced with an apoplectic fit; in another, subject to periodical Epilepsy, it also began with a fit; in a third, who had formerly laboured under an arthritic affection, a severe attack of the gout came on the third day of the fever; in a fourth, subject to hæmoptysis, blood continued to be discharged the whole time of the disease. None, however, suffered more severely nor continued disordered so long as those subject to stomachic complaints. In them, when the disorder was on the decline, cardialgia with all its concomitants, never failed to come on in a very severe degree. Only one (a female) had true pneumonic symptoms, and these yielded to the general mode of treatment employed for the original disorder. Many were affected so slightly as not to require medical assistance.

The following mode of treatment, which was uniformly pursued, succeeded beyond expectation; as out of nearly three hundred cases, consisting of all ages, from a few months old to upwards of eighty years, which came under my care, not one proved fatal. In order to arrest the violent febrile symptoms, the following bolus was immediately administered.

℞. Pulv. ipecac. comp. grs. v. ad viij. calomel. pp. gr. j. ad v. pulv. digital. gr. ß. ad ij. conserv. q. s.

This never failed to procure a remission of the symptoms, the skin became relaxed, the patient felt much relieved, and had some rest. The bolus was repeated at an interval of twelve or twenty-four hours, according to the urgency of the symptoms. But in no case was there occasion to repeat it oftener than four times in the first stage of of the disease; indeed, in general, one or two were sufficient. It may be proper to observe, that when the calomel was omitted, the effect in alleviating the symptoms was very inconsiderable. A few hours after taking the first bolus, two table-spoons full of the following mixture were taken, and repeated every six hours.

℞. Sal. nitri ℈j. ad ʒß. antimon. tartarizat. gr. j. ad ij. spirit. lavend. comp. ʒß. aq. distillat. ℥viij. M.

In general, the first and second dose either cleared the stomach of a quantity of bilious matter, or relaxed the bowels, and sometimes both, with manifest advantage. If, before the exhibition of the mixture, a spontaneous vomiting or diarrhœa had come on, which sometimes happened, a few drops of the tinct. opii were added, by which it produced a greater determination to the skin. The fever was in most cases subdued entirely by the fourth day; in a few instances it continued in a slight degree to the fifth or sixth, when the appetite for food returned. A most distressing dry cough then began, which incessantly harrassed the patient. In some it was removed without much difficulty, but in others various remedies were necessary. The principal which succeeded were small doses of the p. ipecac. comp. conjoined with calomel and digitalis, a composition somewhat similar to the tinct. opii camphorata, but considerably stronger; and tincture of artificial musk. The vertigo and want of appetite, on the decline of the disease, were effectually removed by a weak solution of the antimon. tartarizat. and the stomachic affections were subdued by natron, spt. ammon. comp. colombo, and opiates. The only remedies employed for children were a solution of antimon. tartarizat. with magnesia, and an opiate occasionally. A strict adherence to the antiphlogistic regimen, as it is termed, was particularly proper.

The disease has now entirely disappeared in this place, none having been attacked for these ten days past. From every observation I have been able to make, I am inclined to believe it is not of a contagious nature.

*Chobham, Surry,* I am, &c.

